# In-silico modelling of insulin secretion and pancreatic beta-cell function for clinical applications: is it worth the effort?

**DOI:** 10.3389/fcdhc.2024.1452400

**Published:** 2024-11-04

**Authors:** Andrea Tura, Christian Göbl, Mohamed El-Tanani, Manfredi Rizzo

**Affiliations:** ^1^ CNR Institute of Neuroscience, Padova, Italy; ^2^ Department of Obstetrics and Gynecology, Medical University of Vienna, Vienna, Austria; ^3^ Department of Obstetrics and Gynecology, Medical University of Graz, Graz, Austria; ^4^ College of Pharmacy, Ras Al Khaimah Medical and Health Sciences University, Ras Al Khaimah, United Arab Emirates; ^5^ School of Medicine, Mohammed Bin Rashid University, Dubai, United Arab Emirates; ^6^ Department of Health Promotion, Mother and Child Care, Internal Medicine and Medical Specialties, School of Medicine, University of Palermo, Palermo, Italy

**Keywords:** beta-cell function, in-silico model, mathematical model, glucose sensitivity, rate sensitivity, potentiation factor, insulin secretion, diabetes risk assessment

## 1 Introduction

Recently, there has been ongoing dialogue with clinical researchers about the practical benefits of in-silico mathematical modelling in studying glucose metabolism. In fact, several in-silico models have been developed in such field, as outlined by some review studies (1–4).

Among the different metabolic processes addressed by such models, one relevant is insulin secretion and pancreatic beta-cell function. Indeed, although it is currently known that several factors affect glucose homeostasis (5), the impairment in insulin secretion/beta-cell function, in addition to that of insulin sensitivity, are typically the most important determinants of glycemic control deterioration and possible development of type 2 diabetes. In this opinion article, we will provide considerations about in-silico modelling of beta-cell function.

Some models of beta-cell function describe aspects of such process at molecular or cellular level (6–13). These models are useful to get further insights in relevant molecular/cellular mechanisms, and in addition they can stimulate new experimental research activity in an *in-vitro* context. Other models are instead oriented to describe insulin secretion/beta-cell function at whole body level, and these models are those typically having potential for clinical applications (14–17). In some of the following paragraphs, we focus on the main characteristics and findings of the model by Mari et al. (17). This model has been applied in the clinical context for the analysis of thousands of glucose tolerance tests, including those in wide multicenter projects (such as the IMI-DIRECT Project), focused on longitudinal study of participants with both type 2 diabetes (T2D) (18) and impaired glucose regulation, but also normal glucose tolerance (19). The model by Mari et al. (17) describes three main processes of beta-cell function: the glucose-insulin dose-response relation (“DR” component), the early insulin secretion (“E” component), and the insulin secretion potentiation (“P” component”). We succinctly describe those characteristics in the next section. For brevity, we refer to the model as the DR-E-P model.

## 2 The DR-E-P model of beta-cell function: main characteristics

**Figure 1:**
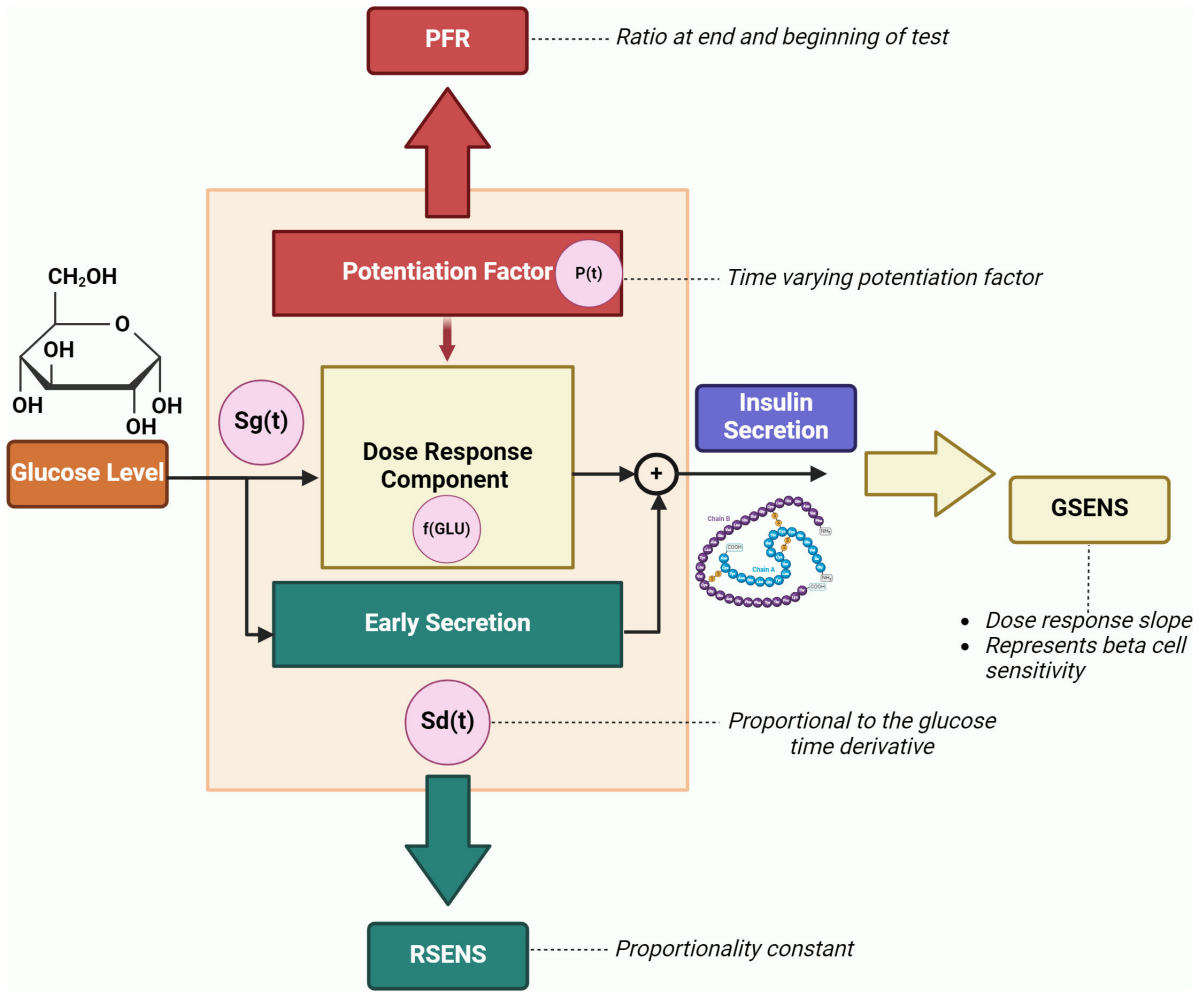
Beta-cell function components and related parameters (GSENS, glucose sensitivity; RSENS, rate sensitivity; PFR, potentiation factor ratio), as described by the DR-E-P model (redrawn from Diabetes Obes Metab 2008;10 Suppl 4:77-87). The graph also reports an additional parameter (secretion at 5 mM glucose), which represents the insulin secretion at a fixed glucose value (typically, 5 mmol/l of glucose level is considered).

The DR-E-P model is mainly applicable to two types of test: the oral glucose tolerance test (OGTT) and the mixed meal test (MMT). These tests are relatively common in the clinical context (at least in the clinical trials), since, as compared to other tests (such as the intravenous glucose tolerance test or the hyperglycemic clamp), they are easier to be performed and determine less burden for both the investigator and the patient. For the DR-E-P model, plasma glucose and C-peptide measures during the OGTT (or the MMT) are required (whereas plasma insulin may be useful but not necessary). In such model, insulin secretion is represented as the sum of two main components, i.e., Sg(t) and Sd(t), where t is the time during the OGTT/MMT. The first component describes the dependence of insulin secretion on absolute plasma glucose levels (GLU), and it is characterized by a nonlinear dose-response function, f(GLU). The mean value of the dose-response slope is named glucose sensitivity (GSENS) and represents the sensitivity to glucose of the beta-cell. The dose-response is modulated by a time-varying potentiation factor, P(t); thus, Sg(t) = P(t)·f(GLU). The ratio of the potentiation factor at the end of the OGTT/MMT to that at the beginning of the test is named PFR (potentiation factor ratio). The second insulin secretion component, Sd(t), describes the dynamic dependence of insulin secretion on the rate of change of glucose levels and it is indicated as the derivative component. Sd(t) is proportional to the glucose time derivative (when the glucose derivative is positive), and the proportionality constant is named rate sensitivity (RSENS). Thus, the model provides three main parameters of beta-cell function: GSENS, RSENS and PFR, which can be estimated in each single OGTT/MMT. **Figure 1** represents the beta-cell function components and related parameters.

## 3 Main qualities of the advanced models of beta-cell function: reliable, robust, refined

The advanced models of beta-cell function mentioned above (14–17) typically share some qualities, which we may summarize in the “triple-R-concept”. They are in fact reliable, robust and refined. We will illustrate these points with regard to the DR-E-P model (17). In fact, in more than twenty years of use, such model has provided evidence of those qualities in several studies. First, the model has shown its reliability. Indeed, the model has demonstrated a remarkable capacity to replicate outcomes from experimental tests that are significantly more complex than the OGTT or the MMT. In the study by Seghieri et al. (20), the model-derived dose-response function was found in agreement with the dose-response derived through a glucose ramp test, in subjects with either normal glucose tolerance or T2D. In addition, the model has been able to provide quantitative and detailed information on beta-cell function that was consistent with what expected by the investigators, such as the progressive beta-cell function deterioration in relation to the worsening of the glycemic control. Indeed, Ferrannini et al. (21) found that GSENS declined in monophasic curvilinear fashion throughout the range of the 2-h plasma glucose. In T2D, impairment in RSENS and in PFR was observed as well.

Furthermore, the DR-E-P model has proven to be robust, meaning that it is typically not prone to outliers or unreliable values, at difference with several “non-model-derived” beta-cell function indices, such as the widely used insulinogenic index or its variants (22, 23). This translates in the higher ability of the model approach to detect even small but specific and clinically relevant changes in the spectrum of beta-cell function. A clear example was seen in one study in women with history of gestational diabetes (GDM) (24), where a group of those women with normal body weight and normal glycemia at 4-6 months after delivery was compared to a group of women without former GDM (acting as control group), with comparable body mass index and glucose tolerance. It was found that the model-derived GSENS was slightly but significantly impaired in former GDM women as compared to the control women. In contrast, the non-model-derived indices (specifically, the insulinogenic index and its variants) failed to show such significant difference between the two groups. Of note, in a subsequent study in those former GDM women, GSENS was found as one of the key predictors of later T2D onset (25).

Moreover, the DR-E-P model is refined, since it provides different parameters of beta-cell function. Although typically the most informative parameter is GSENS, in some studies the importance of assessing different aspects of the beta-cell has clearly emerged. As an example, in one study by Mari et al. (26), in nondiabetic subjects it was found that the beta-cell function at the basal (fasting) level is upregulated by insulin resistance, whereas in dynamic, stimulated conditions the main component of beta-cell function, as represented by GSENS, despite being a key determinant of the glucose tolerance, is unrelated to insulin resistance. These different aspects of beta-cell function cannot be investigated without a model able to dissect the different components of the insulin secretion process. Thus, in the indicated study (26), the model approach was essential to reach the main study conclusion, being that, in the studied population, hyperglycemia mainly resulted from an intrinsic beta-cell defect rather than from inadequate compensation for insulin resistance. It is also worth noting that some of the other models previously mentioned (14–17) share with the DR-E-P model the concept that a deep description of beta-cell function requires different parameters. Especially, the model by Breda et al. (15) includes the beta-cell function parameter named “static sensitivity to glucose, Φ_s_”, that closely resembles the DR-E-P glucose sensitivity, GSENS, as well as the parameter named “dynamic sensitivity to glucose, Φ_d_”, that resembles the DR-E-P rate sensitivity, RSENS.

## 4 Beta-cell function modelling evolution, with an “eye” toward personalized medicine

We should also mention the flexibility of the model approach, since it has potential for being extended. So far, the main DR-E-P model extension has been for the analysis of a pair of tests performed in sequence in the same individual, that is, the OGTT and subsequently the isoglycemic intravenous glucose infusion test (IIGI) (this tests pair also being known as Nauck’s test). The Nauck’s test is in fact considered the best approach for the *in-vivo* assessment of the incretin effect, this meaning the ability of the incretin hormones to enhance glucose-stimulated insulin secretion (27–33). In such DR-E-P model extension (34), OGTT and IIGI are analyzed concomitantly, and this typically provides more robust and reliable analysis as compared to the alternative approach of performing separate analysis of the two tests with the traditional model. This is due to the reason that, with the concomitant analysis allowed by the extended model, the effects of the possible inaccuracies of the IIGI in reproducing the OGTT plasma glucose patterns are softened. In this context, the model approach also allowed exploration of new concepts, such as the OGTT-based beta-cell incretin sensitivity (35).

In addition, further model extension is possible in terms of patient-specific modelling, which leverages the concept of personalized medicine by tailoring in-silico models to individual patients (36). This approach enhances the prediction of disease progression and allows for customized treatment plans, improving clinical outcomes. By integrating patient-specific data, these models provide more accurate and relevant insights into each patient’s unique physiological responses, leading to more effective and targeted therapeutic strategies (37). However, in order to proceed in this direction, it will be necessary to strengthen aspects like model validation and standardization, being crucial for ensuring accuracy and reliability of in-silico models. Further validating these models with experimental data will help confirming their predictive power and applicability. Efforts towards standardizing these models will focus on creating consistent protocols and benchmarks, for enhancing their comparability and reproducibility across different studies and clinical applications, and ultimately fostering greater confidence in their use (38).

## 5 Discussion

In-silico models have significant potential in clinical applications, notably in enhancing diagnostic accuracy and treatment strategies for diabetes (39). Specifically, in-silico models include improved predictions of disease progression and personalized treatment plans that may influence clinical decisions. With regard to the evaluation of beta-cell function, compared to traditional methods, in-silico models offer more comprehensive and dynamic analysis, though requiring specialized expertise and data. These models complement existing methodologies by providing deeper insights into physiological mechanisms and enabling more precise patient-specific evaluations, despite some limitations in routine clinical applicability (37).

It has however to be acknowledged that developing an in-silico model may be a complex task. In addition, even when the model development has been completed, it typically requires specific expertise to be properly used. Furthermore, the model often requires measurement of variables that are not typical of the clinical routine. In the specific case of the beta-cell function model presented in some details in this opinion article, in addition to plasma glucose, plasma C-peptide is required. This may be a limitation in the clinical context, since the diagnostic OGTT (i.e., for possible diagnosis of diabetes) requires only plasma glucose. Moreover, it is worth noting that the discussed model typically requires at least four OGTT (or MMT) samples (possibly including the 30 min sample), that is, not only the diagnostic samples at fasting and at two hours, plus the one hour sample in case of GDM diagnosis. Therefore, the question whether it is worth making the effort of using in-silico models in a clinical context is pertinent, since the indicated drawbacks may prevent models practical applicability. On the other side, as we have illustrated above, an in-silico model can have several qualities. Thus, in our opinion, it may be unreasonable claiming that in-silico models, as the one that we have discussed here, are ready for the routine clinical practice. However, it can be claimed that a model, like the one discussed, can be conveniently applied in clinical investigations, where the pertinent data for using the model are available. Therefore, we believe that using a model is worth the effort, whenever there is the know-how for its use and the required data are available.

Future directions in the in-silico modelling of beta-cell function include the integration of emerging technologies like machine learning and advanced computational methods to enhance model accuracy and applicability (40). Furthermore, ongoing developments will focus on creating more sophisticated models that can incorporate a wider range of physiological data, thus improving predictive power and utility in personalized medicine. These advancements will refine diagnostic tools and treatment strategies, making in-silico models more integral to clinical decision-making.
